# CNF1 Increases Brain Energy Level, Counteracts Neuroinflammatory Markers and Rescues Cognitive Deficits in a Murine Model of Alzheimer's Disease

**DOI:** 10.1371/journal.pone.0065898

**Published:** 2013-05-30

**Authors:** Stefano Loizzo, Roberto Rimondini, Sara Travaglione, Alessia Fabbri, Marco Guidotti, Alberto Ferri, Gabriele Campana, Carla Fiorentini

**Affiliations:** 1 Department of Therapeutic Research and Medicines Evaluation, Istituto Superiore di Sanità, Rome, Italy; 2 Department of Pharmacology, University of Bologna, Bologna, Italy; 3 Department of Veterinary Public Health and Food Safety, Istituto Superiore di Sanità, Rome, Italy; 4 Institute of Cellular Biology and Neurobiology, CNR, Roma, Italy; Nathan Kline Institute and New York University School of Medicine, United States of America

## Abstract

Overexpression of pro-inflammatory cytokines and cellular energy failure are associated with neuroinflammatory disorders, such as Alzheimer's disease. Transgenic mice homozygous for human ApoE4 gene, a well known AD and atherosclerosis animal model, show decreased levels of ATP, increased inflammatory cytokines level and accumulation of beta amyloid in the brain. All these findings are considered responsible for triggering cognitive decline. We have demonstrated that a single administration of the bacterial *E. coli* protein toxin CNF1 to aged apoE4 mice, beside inducing a strong amelioration of both spatial and emotional memory deficits, favored the cell energy restore through an increment of ATP content. This was accompanied by a modulation of cerebral Rho and Rac1 activity. Furthermore, CNF1 decreased the levels of beta amyloid accumulation and interleukin-1β expression in the hippocampus. Altogether, these data suggest that the pharmacological modulation of Rho GTPases by CNF1 can improve memory performances in an animal model of Alzheimer's disease *via* a control of neuroinflammation and a rescue of systemic energy homeostasis.

## Introduction

The apolipoproteins E (apoE) are cholesterol transporters of high importance for neuronal plasticity, glucose utilization, mitochondrial functions and modulation of inflammation [1], [2]. However, individuals homozygous for one dysfunctional variant of apoE, the apoE4 allele, are known to be at major genetic risk for developing atherosclerosis [3] and sporadic Alzheimer's Disease (AD) [4], [5]. ApoE4 allele is also associated with an enhanced risk or severity of several other neurodegenerative disorders [6], with a faster progression of certain neuromuscular diseases, including diabetic neuropathy and human immunodeficiency viral neuropathy [7], and with neuroinflammation, in pathologies like multiple sclerosis [8], Parkinson's disease [9], [10] and those neurological diseases involving excitotoxic mechanisms [11]. In fact, apoE4 genotype has a profound influence on the extent of disease-related synaptic deterioration due to its dumping effects on dendritic growth [12], and therefore, on neuronal plasticity. In addition, apoE4 uniquely undergoes neuron-specific proteolysis, resulting in bioactive toxic fragments that enter the cytosol, alter the cytoskeleton and disrupt mitochondrial energy balance [13]. Transgenic mice homozygous for human apoE4, show mitochondrial dysfunction, with decreased production of metabolic energy in terms of decreased levels of adenosine triphosphate (ATP) and show accumulation of beta amyloid (Aβ or Abeta) in the brain [14], [15], [16]. Moreover, pro-inflammatory cytokines, important markers of the inflammatory component of apoE4 [17] and, in general, of AD [18], are strictly connected with brain energy metabolism [19].

Recently, we have demonstrated that an *Escherichia coli* protein toxin, named Cytotoxic Necrotizing Factor 1 (CNF1), improves the mitochondrial activity and regulates pro-inflammatory cytokines levels in a mouse model of Rett's syndrome [20]. These effects, accompained by a long-lasting amelioration of cognitive performances, were strictly Rho GTPases-dependent. The Rho GTPases, ubiquitously expressed molecular switches that cycle between a GDP-bound inactive and a GTP-bound active state in eukaryotic cells, encompass the three subfamilies Rho, Rac and Cdc42 that control different signalling pathways. All of them are constitutively activated by CNF1 through deamidation of a critical glutamine residue that lock them in their activated, GTP-bound state [21], [22]. The threeshold of this activation is subsequently attenuated because high levels of activated Rho GTPases are recognized by cells that ubiquitinate and degrade them to more physiological levels [23]. The ability of Rho GTPases to control actin polymerization [24], plays important roles in the morphogenesis of the dendritic spines in the brain [25], [26], [27] as well as in the synaptic plasticity [28], [29], [30], [31], [32], [33]. Our previous studies showed the ability of CNF1 to trigger structural remodelling and functional plasticity in rodents [34], [35]. Deficits in neuronal plasticity have been reported in several pathologies of the central nervous system (CNS) characterized by energy and cognitive deficiencies, including Rett syndrome [36] and AD [37]. Very recently, it has been reported that CNF1 can ameliorate cognitive performances in four-month old TgCRND8 mice, an AD model with early-onset Aβ deposits [38], thus confirming our previous hypothesis [39]. It remains totally unexplored, however, the mechanism by which CNF1 can improve the AD-linked behavioural deficits, and whether CNF1 can counteract the presence of Aβ tangles that are considered the main cause of cognitive impairment.

To address these questions, we used clearly symptomatic (12 months old) apoE4 hemizygous (hz) male mice that show, on a normal diet, altered relative quantities of different plasma lipoprotein particles, and delayed clearance of very low density lipoprotein (vLDL) particles, with only half the clearance rate observed in the apoE3 targeted replacement mice [40]. Furthermore, apoE4 mice, if compared to apoE3, are characterized by a more rapid, age-related cognitive decline associated with neuroinflammatory responses [41]. Finally, apoE4 mouse model is considered useful for studying the role of human apoE polymorphism in atherosclerosis, lipid metabolism [3] and Alzheimer's disease [2].

Using this animal model, we found that a single dose of intracerebroventricular (icv) administration of CNF1 improved spatial and emotional memory and modified the cell energy, in terms of ATP content, as well as the levels of Aβ and of the pro-inflammatory cytokine IL-1β. It is noteworthy, that all these aspects are directly or indirectly regulated by Rho GTPases [41], [43] and are considered crucial markers in AD mouse models [14], [15], [16], [17], [18]. Taken altogether, we can speculate that the striking improvement of the cognitive defects in CNF1-treated mice is most probably linked, *via* the pharmacological modulation of Rho GTPase signaling, to a restoration of physiological energy levels and to anti-inflammatory processes.

## Results

### CNF1 improves learning and memory retention (spatial and non-spatial) in apoE4 mice

The first step of our investigation was to verify whether CNF1 could improve learning and memory in aged apoE4 mice (12-month old) that present advanced cognition deficits [44], [45]. To answer this question, we have used Morris water test for reference memory (spatial memory) and Passive Avoidance for emotional memory (non-spatial memory).

**Figure 1:**
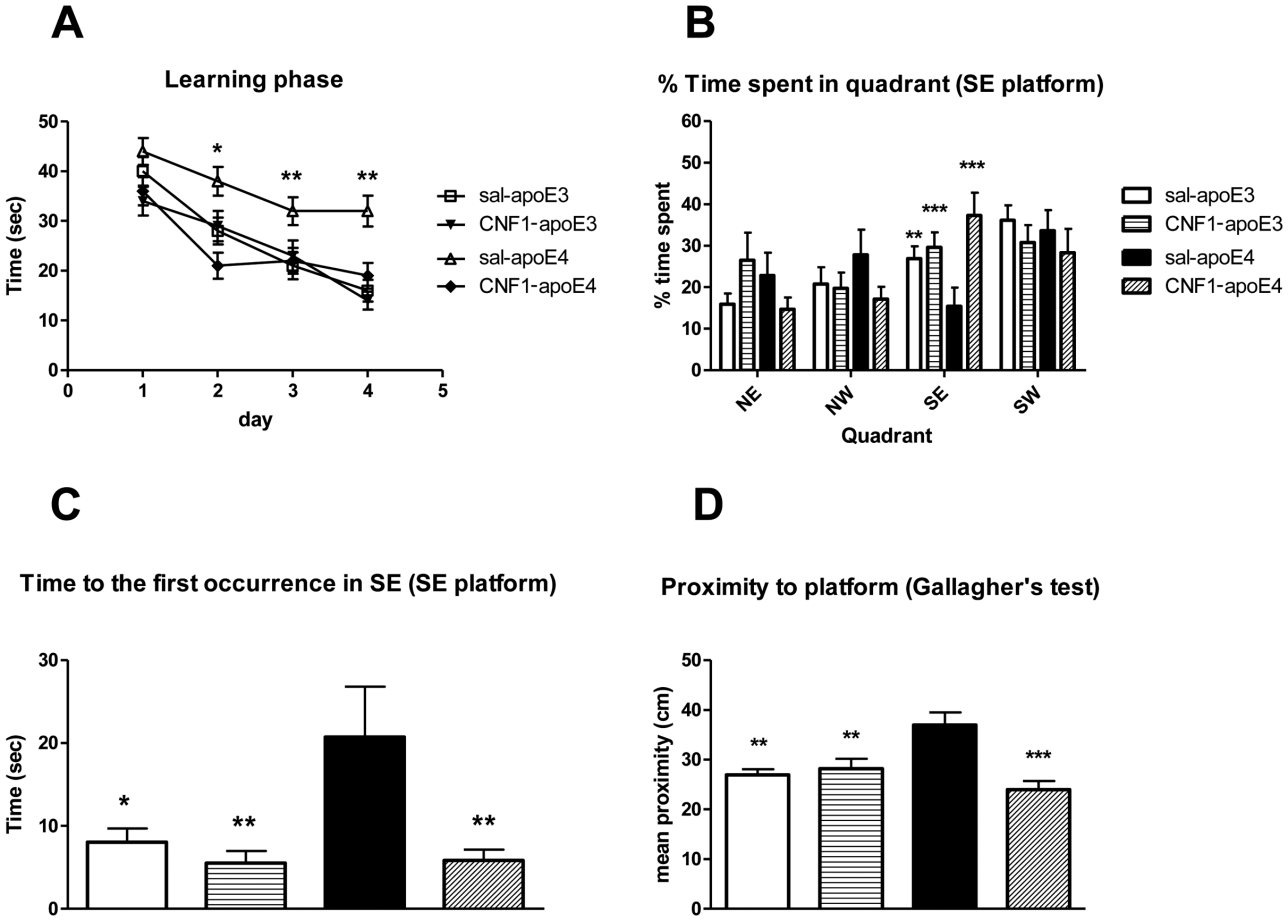
Effects of apoE genotype and CNF1 treatment in the Morris water maze test. Morris water maze performance in 12 month-old apoE3 and apoE4 (13–14 mice/group) with a intraventricular saline or CNF1 (0.1 nM) injection. A. Learning curve for training to find the hidden platform in the Morris water maze. The latency to find the platform in the 4 sessions of each day was averaged in order to obtain one value/animal/day. Mean ± standard error of the mean (SEM) values are shown. Statistical analysis of the overall learning curve was performed by means of repeated measures two-way ANOVA, for detailed statistical analysis, see Results. B, C and D. Probe test performance. Quadrants are designated as follows: SE  =  the quadrant of the maze that contained the submerged escape platform during training, NE  =  the quadrant of the maze to the left of the training quadrant, SW  =  the quadrant of the maze to the right of the training quadrant. NW  =  the quadrant of the maze directly opposite to the training quadrant. B. % Time spent in the quadrant where the platform was placed during the training phase. C. Latency to reach the platform position for the first time. D. Gallangher's proximity test (mean proximity to former platform location). Data are shown as mean ± SEM. For detailed statistical analysis, see Results. * = p<0.05, ** = p<0.001 and *** = p<0.0001 saline-apoE4 vs. all other groups.

Saline or CNF1 treated apoE4 (n = 13–14) and apoE3 (n = 13–14) mice were tested for their ability to find a hidden platform in the Morris water maze for 4 days and were subjected to the probe test 24 h after the last training session. Analysis of the learning curve (Figure 1A) by repeated measures, two-way ANOVA showed a significant genotype x treatment interaction and genotype x treatment x time as well as a significant main effect of time (indicating an overall improved performance with time) and treatment [Time: F_(3,141)_ = 32.57, p<0.001; genotype: F_(1, 50)_ = 11.47, p = 0.0014; treatment: F_(1, 50)_ = 14.91, p = 0.00034; genotype x treatment: F_(1, 50)_ = 4.72, p = 0.035; genotype x treatment x time F_(3, 150)_ = 3.42, p = 0.018]. In view of the significant interaction, we then performed a repeated measure, one-way ANOVA on the 4 groups, showing that the performance of saline apoE4 mice was significantly worse than that of all other groups, that in turn were not significantly different between them [F_(12, 130)_ = 3,9276, p = 0.00004; posthoc Fisher LSD test: day 1 no significance between all groups; day 2 saline apoE4 vs. CNF1 apoE4 p = 0.001, vs. apoE3 p = 0.02, vs. CNF1 apoE3 p = 0.00006 all other comparisons NS; day 3 saline apoE4 vs. CNF1 apoE4 p = 0.05, vs. apoE3 p = 0.01, all other comparisons NS; day 4 saline apoE4 vs. CNF1 apoE4 p = 0.00000, vs. apoE3 p = 0.00000, vs. CNF1 apoE3 p = 0.00000 all other comparisons NS] (to get an overview summary, the statistical analysis data were grouped into the supplementary results section. See Tables S1 and S2 in File S1).

On day 5, mice underwent the probe test. The percentage of time spent by the mouse in the quadrant (south-east, SE) where the platform was placed during the training phase is considered as an index of spatial memory [46] (Figure 1B). The analysis of behavior in the probe test was carried out by two-way ANOVA and showed a significant interaction genotype x treatment [F_(1, 50)_ = 4.450, p = 0.035] and a significant main effect for Treatment [F_(1, 50)_ = 9.274, p = 0.0037] but no main effect for genotype [F_(1, 50)_ = 0.094, NS] for the percentage of time spent in the correct quadrant (Figure 1B). A one-way ANOVA revealed that % time spent in SE quadrant of saline-treated apoE4 groups was significantly decreased compared to all other groups that did not differ between them [F_(3, 50)_ = 54.624, p = 0.0064; posthoc LSD test: saline apoE4 vs. CNF1 apoE4 p = 0.0006, vs. saline apoE3 p = 0.05, vs. CNF1 apoE3 p = 0.021, all other comparisons NS] (Tables S3 and S4 in File S1). Therefore, the genotype main effect at the two-way ANOVA is probably not relevant. Similar results were obtained when we analysed the latency of first entrance in SE quadrant (Figure 1C). One-way ANOVA showed a significant main effect [F_(3, 50)_ = 3.0323, p = 0.037] and Fisher LSD post-hoc analysis revealed that latency of the first occurrence in saline apoE4 was increased if compared to all other groups those did not differ between them (saline apoE4 vs. CNF1 apoE4 p = 0.01, vs. saline apoE3 p = 0.02, vs. CNF1 apoE3 p = 0.02, all other comparisons NS) (Tables S5 and S6 in File S1). Since a recent study concluded that, in the mouse, ‘mean proximity to former platform location (Gallangher's proximity test)’ is more sensitive to group differences than other measures (zonal or quadrant durations and crossings [47]), we used this quantitative analysis that analyze the mean swim distance from former platform position (Figure 1D). Gallangher's proximity test showed that CNF1-apoE4 mice swam more distant (mean 38,21±2,53 cm) compare to apoE3 (mean 26,13±1,18 cm), CNF1-apoE3 (mean 28,37±1,98 cm) and CNF1-apoE4 (mean 23,75±1,72 cm). One-way ANOVA showed a significant main effect [F_(3, 50)_ = 4.648, p = 0.0068] and Fisher LSD post-hoc analysis revealed that mean the proximity in saline apoE4 was increased if compared to all other groups that did not differ between them (saline apoE4 vs. CNF1 apoE4 p = 0.0006, vs. saline apoE3 p = 0.01, vs. CNF1 apoE3 p = 0.01, all other comparisons NS) (Tables S7 and S8 in File S1). No differences in swimming distance and velocity were observed (data not shown).

**Figure 2:**
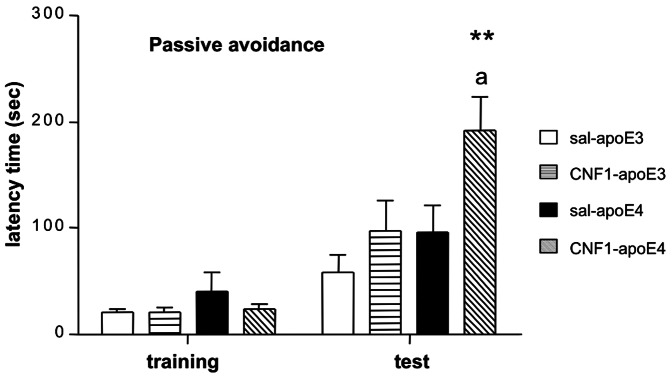
Effects of apoE genotype and CNF1 treatment in the passive avoidance test. Data are shown as latency time to enter the dark compartment on the training day (day 1) and the probe day (day 2) of the test. No differences between experimental groups were observed during training days. CNF1-treated apoE4 mice showed an increase in the latency time to re-enter the dark compartment after the electric shock (p = 0.004) compared to all other groups. Results are expressed as mean± SEM, n = 13–14 animals per group (**p<0.002 compared to saline-apoE3, a p<0.048 compared to saline-apoE4 mice).

To study the possible effects of CNF1 on no-spatial memory, we used the passive avoidance test (PA). PA is a test of rapid one-trial learning, where an animal is conditioned with a single aversive event and is later tested for recollection of that experience. Avoidance tasks are technically similar to cued and contextual conditioning and the stimulus is a mild footshock; the response is avoidance of the location in which the footshock was received. PA tasks require the mouse to refrain from entering the chamber in which the aversive stimulus was previously delivered. Results of PA test are shown in the graph of Figure 2, which reports the latency time to enter the dark compartment on the first day (training) and on the second day (test) of the behavioral procedure. On the first day, all the groups showed similar step-trough latencies, indicating no differences in locomotor activity and anxiety-like behavior induced by the novel stress envirorment among the experimental groups. After 24 h (day 2, test), animals were re-placed in the test apparatus to test their memory. Mice of all genotypes and both treatment learned the task as they showed a clear increase in latency to enter the dark compartment between the two sessions. During this second day, one-way ANOVA revealed a statistical significant differences induced by the treatments (main effect: F_(3, 50)_ = 4.9547, p = 0.00434) in the latency time to re-enter the dark compartment after the electric shock. Post-hoc analysis (Tukey HSD test) showed significant differences when comparing CNF1-apoE4 with apoE3 (p = 0.002) and with apoE4 (p = 0.048). A tendency toward a significance difference (p = 0.059) was found when CNF1-apoE4 was compared with CNF1-apoE3. No significance differences were found between saline-apo3 and saline-apoE4 (Table S9 in File S1). At behavioral observation, no long term side effects were detected during the experimental phase (30 days).

These results confirm the property of CNF1 to improve learning and memory performances in a validated AD animal models.

### CNF1 does not induced any changes in anxiety-like behavior in apoE4 and apoE3 mice

In order to verify if the previous observed differences between apoE4 apoE3 mice, treated with saline or CNF1, were due to the experimental stresseful conditions of the spatial and no-spatial memory tests performed, we looked at possible changes in anxiety-like behavior.

We analyzed the behavior of saline and CNF1-treated apoE3 and apoE4 mice on Elevated Plus Maze (EPM) and Open Field (OF). As shown in Table 1, data from EPM did not reveal any statistical difference among the experimental groups [Total entries (TE): treatment effects F_(1, 50)_ = 1.2894, p = 0.26157, NS, genotype effect F_(1, 50)_ = 1.3161, p = 0.25675, NS; genotype x treatment interaction effect F_(1, 50)_ = 1.3868, p = 0.24452, NS; % open entries (%OE) treatment effects F(1, 50) = 0.21216, p = 0.64708, NS; genotype effect F_(1, 50)_ = 0.34155, p = 0.56156, NS; genotype x treatment interaction effect F_(1, 50)_ = 0.39574, p = 0.53216, NS; % open time (%OT) treatment effects F_(1, 50)_ = 1.3952, p = 0.24312, NS, genotype effect F_(1, 50)_ = 0.59611, p = 0.44370, NS; genotype x treatment interaction effect F_(1, 50)_ = 0.38472, p = 0.53790, NS] (Tables S10, S11 and S12 in File S1). Furthermore, no differences were observed in OF data (data not shown). At behavioral observation, no long term side effects were detected during the experimental phase (30 days).

**Table 1:** Effect of acute saline or CNF1 *icv* with regards to Elevated Plus Maze (EPM) behavior.

Treatment and Strain	Total Entries (TE)	% Open Time (%OT)	% Open Entries (%OT)
Saline-apoE3	28.35±9.83	34.73±15.67	48.19±11.49
CNF1-apoE3	22.92±7.29	30.25±17.78	47.72±10.64
Saline-apoE4	28.28±7.01	32.14±14.52	44.78±9.49
CNF1-apoE4	28.38±9.93	29.55±13.42	47.84±9.52

Data (mean±SEM) are shown for apoE3 or apoE4 acutely treated with CNF1 (0.1 nM *icv*) or saline (*icv*). CNF1 or saline treatment did not induce any changes in anxiety like-behavior. For detailed statistical analysis, see Results.

These results indicate that neither treatments nor the genotype influence anxiety-like behavoir in apoE4 and apoE3 mice, thus excluding the involvement of anxiety in the observed differences in memory-related behavior among the experimental groups.

### CNF1 counteracts the hyper-activation of Rho GTPases in the hippocampus of apoE4 mice

CNF1 has been so far indicated only as an activator of Rho GTPases, in the total brain [39] and in single, specific areas, as Periaqueductal gray (PAG) area [34] and the visual cortex [35] of wild-type rodents. The activity of CNF1 on Rho and Rac GTPases is herein investigated, for the first time, in the hippocampus and the frontal cortex of a pathological mouse model, the apoE4 mice. As control, the neuroprotective variant apoE3 was used.

**Figure 3:**
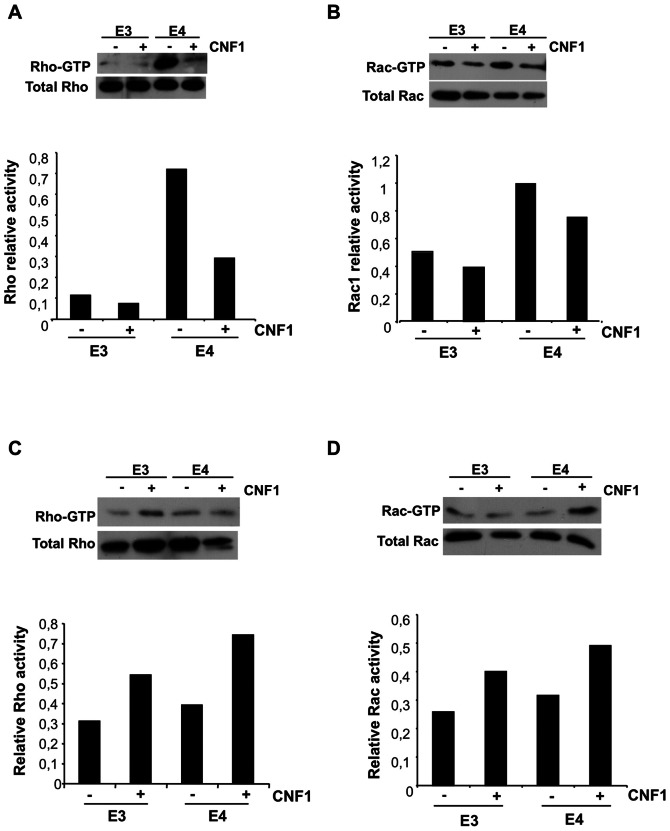
Modulation of Rho GTPases' activity by CNF1 in hippocampus and frontal cortex. Immunoblots showing representative Pull down experiments (upper panels) were normalized as a function the total proteins loaded in the assay and the results expressed as relative activity (histograms, bottom panels). A, B. Pull down of Rho-GTP (A) and Rac-GTP (B) in hippocampus. C, D. Pull down of Rho-GTP (C) and Rac-GTP (D) in frontal cortex.

We tested 6 mice per group. Figure 3 shows the results of a representative experiment. We found that the Rho protein was hyper-activated in the hippocampus of apoE4 mice with respect to control apoE3 mice (Figure 3A) and, interestingly, CNF1 was able to reduce the amount of Rho-GTP in apoE4 mice, while having no significant effects on Rho activation in healthy mice (apoE3). Also Rac1 was hyperactivated in the hippocampus of apoE4 with respect to apoE3 mice, although to a lesser extent (Figure 3B). In this case, however, CNF1 induced only a slight decrease in Rac1-GTP levels either in apoE3 or in apoE4 mice. The toxin capacity to reduce the activity of these regulatory proteins to physiological levels in cells [23] could explain the observed drop of Rho-GTP levels in the hippocampus. By contrast, in the cortex area no differences were observed in Rho and Rac1 activation state between apoE4 and apoE3 mice, and CNF1 provoked an increase in Rho and Rac1 activation in both experimental groups (Figure 3C and D, respectively).

These results evidence that CNF1 not only induces a different response in different areas of the brain, but also it is able to counteract the hyper-activation of cerebral Rho proteins, a finding that has never been reported previously in experimental animals challenged with CNF1.

### CNF1 modulates ATP levels in the hippocampus and cortex

**Figure 4:**
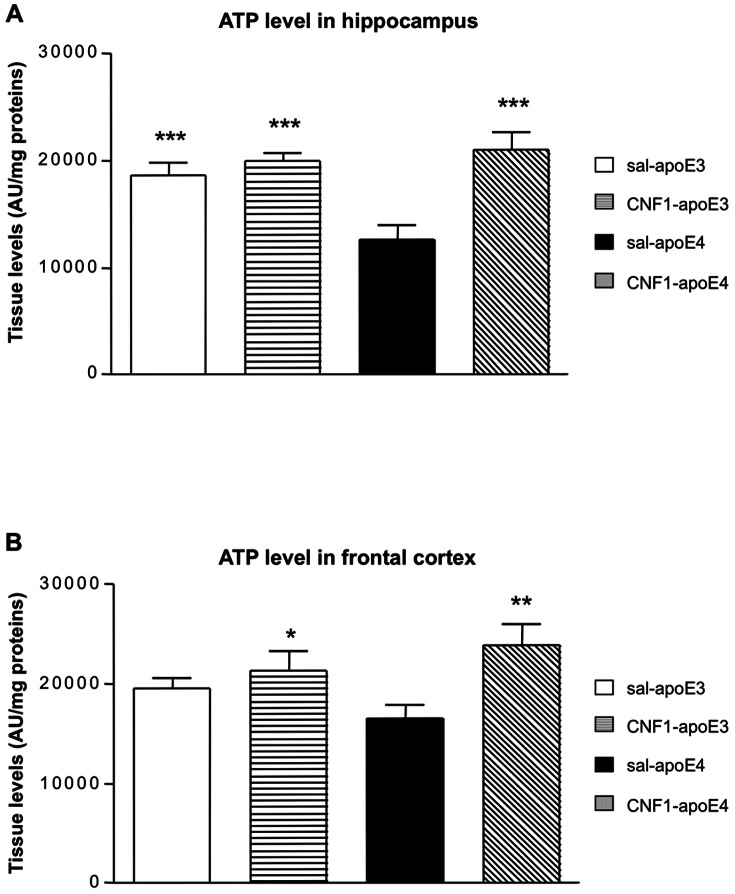
Effect of CNF1 on cerebral ATP levels in apoE4 and apoE3 mice. Hippocampal (Hip, A) and cortical homogenates samples (Cx, B) from apoE3 and apoE4 mice treated with saline or CNF1 were analyzed by ELISA immunoblotting. ATP levels were increased in saline-apoE3, CNF1-apoE4, CNF1-apoE3 mice compared to apoE4 mice (*** p<0.0001). Data are shown as mean±SEM (AU/mg proteins); n = 7 per group.

We have previously shown that the Rho GTPases, activated by CNF1, are involved in the regulation of mitochondrial functionality in mouse brain [20]. AD and atherosclerosis are characterized by mitochondrial dysfunctions and energy failure that can damage neurons through increased production of reactive oxygen species (ROS), ATP depletion and activation of cell death processes [48], [49]. To verify whether CNF1 can modulate mitochondrial activity, we analyzed ATP levels in the hippocampus (Figure 4A) and frontal cortex (Figure 4B) of apoE4 and apoE3 mice treated with saline or CNF1. Statistical analysis of the data obtained in the hippocampus (Figure 4A) by factorial two-way ANOVA showed a significant effect of genotype [F_(1, 31)_ = 4.3068, p = 0.0463], treatments [F_(1, 31)_ = 17.004, p = 0.00026] and of the interaction treatment x genotype [F_(1; 31)_ = 9.274, p = 0.004]. Post hoc LSD revealed that hippocampal ATP levels were significant lower in saline-apoE4 compare to saline-apoE3 (p = 0.0008), CNF1-apoE4 (p = 0,00004) and CNF1-apoE3 (p = 0.00004) (Table S13 in File S1).

In frontal cortex (Figure 4B), no significant genotype effect was found [F_(1, 20)_ = 0.0298, p = 0.8]. Anyway, significant treatments and treatment x genotype interactions were revealed [treatments effects F_(1, 20)_ = 22.685, p = 0.00012; treatments x genotype F_(1, 20)_ = 4.824, p = 0,04001]. Post-hoc LSD analysis showed that ATP levels in cortex were significant lower in saline-apoE4 compared to CNF1-apoE4 (p = 0.00049) and CNF1-apoE3 (p = 0.002). ) (Table S14 in File S1).

Therefore, we can hypothesize that the ability of CNF1 to remodel the mitochondrial network [50], accompanied by increased ATP levels, may favor the cell energy restore and the correct neuronal function.

### CNF1 modulates IL-1β neuroinflammatory cytokine level in hippocampus of apoE4 mice

**Figure 5:**
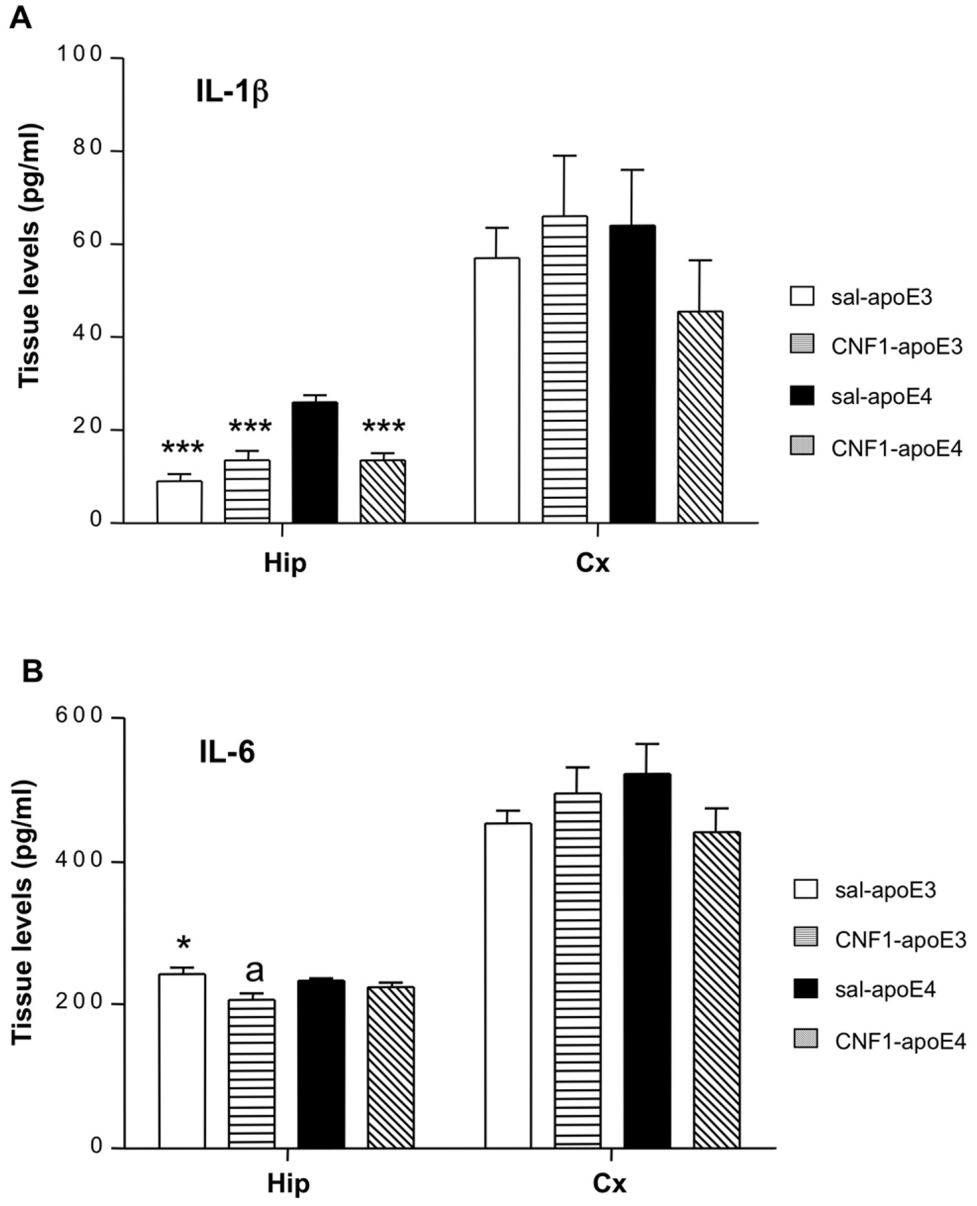
CNF1 treatment diminished IL-1β in the hippocampus of apoE4 mice. Hippocampal (Hip) and cortical (Cx) samples from apoE3 and apoE4 mice, treated with saline or CNF1, were analyzed by ELISA. A. Graph showing IL-1β levels: CNF1 was able to significantly decrease IL-1β levels in the hippocampus of apoE4 mice to values comparable to those of ApoE3 mice, while having no effect in apoE3 healthy mice. B. Graph showing IL-6 levels: CNF1 was not able to mofify IL-6 amount either in hippocampus or in frontal cortex of apoE3 and apoE4 mice. Data are shown as mean ± SEM (pg/ml). Sal-apoE3 mice were used as reference. *p<0.05 vs sal-apoE4; ***p<0.001 vs sal-apoE4, a p<0.05 sal-apoE3 vs CNF1-apoE3; n = 7 per group.

An increasing number of studies implicates the overexpression of pro-inflammatory cytokines in the pathogenesis of apoE4-associated neurodegenerative (such as AD and Parkinson disease), and autoimmune disorders (such as multiple sclerosis) [51]. Recently, we have demonstrated that CNF1 can significantly decrease the levels of IL-1β in pure astrocytic cultures [52] and the level of IL-6 in a mouse model of Rett's syndrome [20]. To determine whether the genotype and the treatment affect the amount of brain pro-inflammatory cytokines in apoE3 and apoE4 mice, IL-1β and IL-6 levels were measured by ELISA in hippocampal and cortical homogenates (Figure 5).

Hippocampal IL-1β levels are shown in Figure 5A. Factorial two-way ANOVA demonstrated a significant strain effect, treatments effect and interactions treatment x genotype [genotype effects F_(1, 22)_ = 25.727, p = 0.00004; treatments effects F_(1, 22)_ = 5,8514, p = 0.024; interaction genotype x treatments F_(1, 22)_ = 25,757, p = 0.00004]. Post hoc LSD analysis revealed that IL-1β levels in sal-apoE4 are significantly higher compared to sal-apoE3 (p = 0.0000), CNF1-apoE4 (p = 0.000026) and CNF1-apoE3 (p = 0.000026). No significant effects of CNF1 were observed in cortical homogenates [genotype effect F_(1, 20)_ = 0.37281, p = 0.54836 NS; treatment effect F_(1, 20)_ = 0.20636, p = 0.65453, NS; interaction genotype x treatment effect F_(1, 20)_ = 1.6018, p = 0.22019, NS] (Figure 5A; Tables S15 and S16 in File S1).

As concerns IL-6, factorial two-way ANOVA revealed a significant treatment effect [treatment effect F_(1, 21)_ = 11,204, p = 0.00305] in hippocampus (Figure 5B). No other significant effects were found [genotype effect effect F_(1, 21)_ = 0.35621, p = 0.55700, NS; interaction genotype x treatment effect F_(1, 21)_ = 2,6057, p = 0.12141]. Post hoc LSD test revealed a significant difference between saline-apoE4 vs sal-apoE3 (p = 0.012) and saline-apoE3 vs CNF1-apoE3 (p = 0.0017). No significant differences were found in the cortex (Figure 5B) in the four experimental animal groups [genotype effect F_(1, 20)_ = 0.05564, p = 0.81592 NS; treatment effect F_(1, 20)_ = 0.40071, p = 0.53389, NS; interaction genotype x treatment effect F(1, 20) = 3.4200, p = 0.07925, NS] (Tables S17 and S18 in File S1).

The above results clearly indicate that CNF1 is able to contrast the up-regulation of the pro-inflammatory cytokine IL-1β, which characterizes the neuroinflammatory condition of apoE4 mice. This finding is relevant since pathophysiological levels of IL-1β are known to produce detrimental effects on neuroinflammation and on memory functioning [18].

### CNF1 prevents Aβ accumulation in hippocampus of apoE4 mice

**Figure 6:**
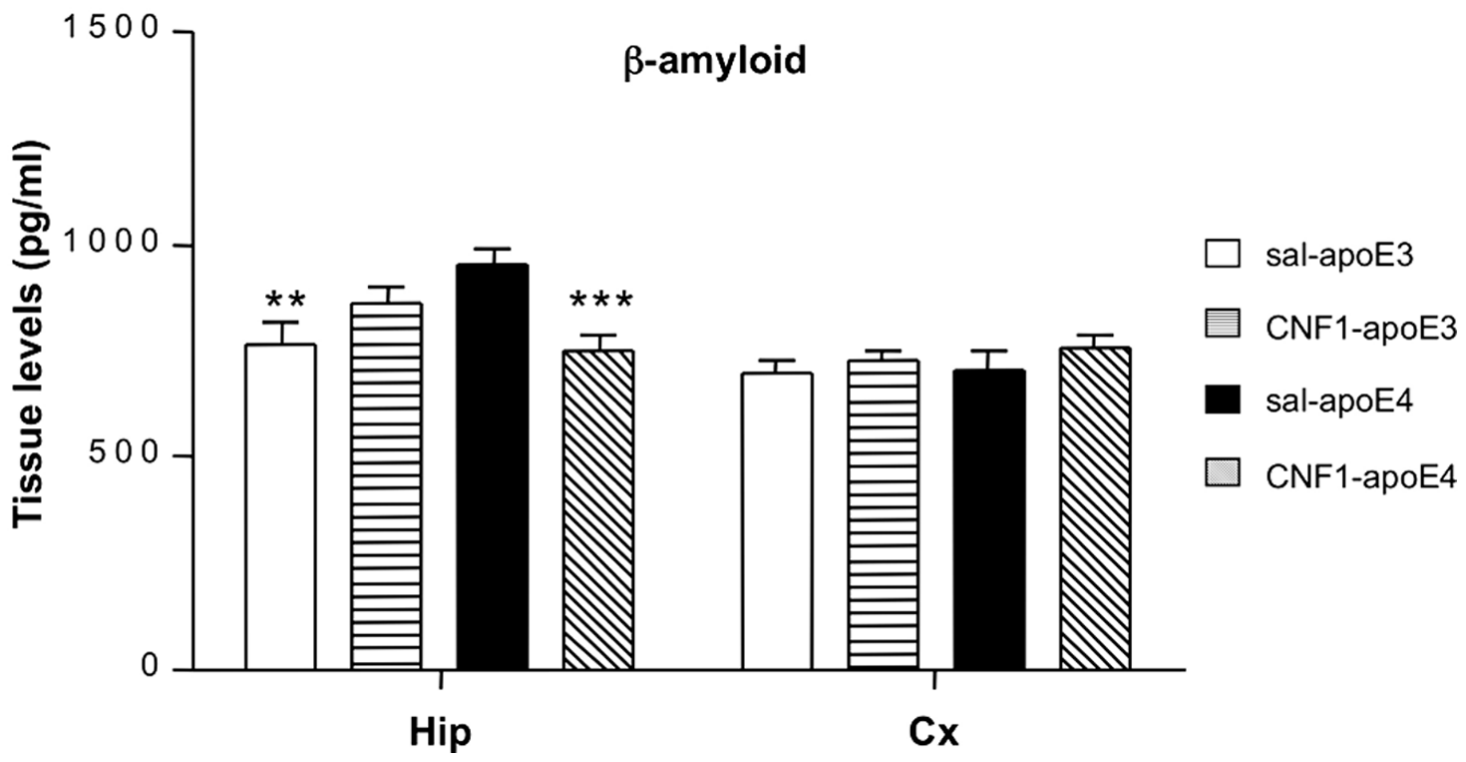
CNF1 treatment diminished Aβ42 levels in the hippocampus of apoE4 mice. Hippocampal (Hip) and frontal cortex (Cx) samples from apoE3 and apoE4 mice treated with saline or CNF1 were analyzed by ELISA. CNF1 treatment in apoE4 resulted in a significant decrease in the levels of Aβ42 to apoE3 level. No CNF1 effect was observed in apoE3 mice. CNF1 did not induce significant changes in Aβ42 levels in apoE3 and apoE4 cortex. Data are shown as mean ± SEM (pg/ml). Sal-ApoE3 mice were used as reference. *p<0.05 vs sal-apoE4; ***p<0.001 *vs* sal-apoE4, a p<0.05 sal-apoE3 *vs* CNF1-apoE3; n = 7 per group.

Aβ deposition is one of the most important AD markers and, interestingly, increased release of the “longer forms” of Aβ peptide, Aβ42 and Aβ43, occurs in individuals expressing apoE4 allele, leading to abnormal deposition of Aβ, and the consequent associated brain neurotoxicity [53]. Thus, we decided to verify whether CNF1 could counteract Aβ42, the more fibrillogenic form associated with disease state of apoE4 mouse [54]. As shown in the Figure 6, factorial two-way ANOVA revealed a significant interaction beween treatment and genotype [genotype x treatment interaction effect F_(1, 21)_ = 15.255, p = 0.00081; genotype effect F_(1, 21)_ = 0.94182, p = 0.34286, NS; Treatment effect F(1, 21) = 2.0886, p = 0.16316, NS]. Post hoc LSD analysis showed that the hippocampus of apoE4 mice was characterized by a higher Aβ42 basal level with respect to apoE3 (p = 0.0027), while no difference was detected between apoE4 and apoE3 cortex samples (Figure 6). Interestingly, CNF1 was able to significantly reduce the Aβ level measured in the hippocampus of apoE4 mice down to a value close to apoE3 mice levels. By contrast, CNF1 treatment caused no significant changes in the level of Aβ in apoE3 hippocampi (Figure 6). Furthermore, no differences in Aβ42 levels were found in the frontal cortex of the four experimental animal groups [genotype x treatment interaction effect F_(1, 20)_ = 0.212, p = 0.649; genotype effect F_(1, 20)_ = 0.3309, p = 0.571, NS; treatment effect F_(1, 20)_ = 1.8071, p = 0.1939, NS] (Figure 6). A summary of statistical analysis is in Tables S19 and S20 in File S1.

In our experimental conditions, CNF1 acts only on altered Aβ42 value detected in the hippocampus of apoE4 mice, suggesting a modulatory effect, rather than a straightforward inhibitory effect.

## Discussion

The present study was designed to explore the effects of the Rho GTPases' modulator CNF1 on aspects that are behind learning and memory retention. Particularly, we investigated the involvement of energy homeostasis and neuroinflammation in the response to CNF1 by the isoforme variant human apoE4 mouse, a validated sporadic AD and atheroschlerosis murine model, and by its neuroprotective variant apoE3.

Even though the association of the ε4 allele of ApoE with AD was demonstrated two decades ago [4], yet the underlying mechanisms are not completely clarified. The fact that the presence of ApoE4 *per se* is not sufficient for the development of AD, suggests that it might interact with other factors (genetic, environmental or biochemical) to participate in the pathogenesis of cognitive decline and dementia. Data from clinical, epidemiological, and preclinical studies have suggested that individuals with dysfunction in energy balance and a neuroinflammation state bear an increased risk of AD [55]. In fact, it is well known that compromised mitochondrial bioenergetics lead to over-production and mitochondrial accumulation of Aβ, which is coupled to oxidative stress. The prevailing “amyloid cascade” hypothesis emphasizes the neurotoxic characteristics of Aβ as the main contributor to disease progression. However, rather than being the cause of the disease, Aβ may be considered as a reactive product that arise from increased vulnerability to genetic and environmental risk factors as a function of aging [56], [57], [58]. Moreover, candidate drugs that directly target amyloid pathways, [59], [60], failed to achieve efficacy in recent clinical trials, indicating the limited therapeutic value of amyloid-specific strategies. Increasing evidence suggests that Alzheimer's disease is a multifaceted disease that could at least in part be attributed to a decline in mitochondrial function and altered brain metabolic activity [61], [62].

In this paper we report that a single icv injection of CNF1 improved memory processes in 12-month old apoE4 mice compared with apoE3 mice. To perform studies on memory, animals were behaviorally tested first in the Morris water maze (MWM) and subsequently in the PA test. This temporal order took into account the level of stress associated with each procedure [54]. Using MWM test we show that, during the acquisition phase, the escape latencies to find the hidden platform were shorter in saline-treated apoE3 (sal-apoE3) mice compared to saline-treated apoE4 (sal-apoE4) mice, indicating a clear impairment in learning skills. Anyway, apoE4 exposed to CNF1 (CNF1-apoE4) showed an improvement during the acquisition phase and the escape latencies were comparable with those of the no-pathological control strain apoE3. Furthermore, while our behavioral results revealed that sal-apoE4 had a clear impairment in memory retention, CNF1 treatment improved apoE4 mice retention. It is noteworthy that CNF1 did not alter performances in apoE3 animals.

The PA test is an amygdale-dependent test which evaluates the ability of mice to learn and to retain an associative rule [63]. CNF1 apoE4-treated mice showed definite longer latencies to enter the dark compartment during the test session of the passive avoidance test (Fig. 2). As a result, these animals were able to learn or to retain the rule: black compartment is equal to electrical shock. The results from the passive avoidance test suggest that at 12 months of age apoE4 mice have not impaired passive avoidance memory retention compare to apoE3. These results are in line with previous experimental evidence [64] where it has been shown that there was no genotype on any classical measure of passive avoidance response, such as latency to enter the dark compartment during acquisition and retention sessions. On the contrary, CNF1-apoE4 retained contextual fear indicating that amygdala-dependent long-term memory is affected by CNF1. Taken toghether these data could indicate that CNF1 ameliorate no-spatial memory performances only in pathological condition (presence of apoE4 allele).

Since clinical [65] and experimental [66] evidences show that the presence of apoE4 correlated positively with an anxious state, we studied the effects of CNF1 on anxiety-like behavior tracts. These studies were performed prior WM and PA taking into account the level of stress associated with each procedure [67]. Our data show that sal-apoE4 did not increase measures of anxiety when compared to sal-apoE3 mice and the icv treatment with CNF1 did not induce any changes in EPM and OF performances. Furthermore, there were no group differences in total locomotor activity measurements both in MWM, EPM and OF, indicating that the differences observed during MWM and PA test do not reflect differences in activity levels or locomotor impairments. It is well known that dysfunctions in motor neurons, cerebellum and spinal cord will reflect in slower swim speed, random swim pathway [68].

To unravel the mechanisms underlying the effects of CNF1 on cognition in apoE4 mice, we performed molecular studies on the hippocampus and frontal cortex, focusing on different markers involved in memory, energy and neuroinflammation processes. We found, interestingly, that there is a genotype specificity in hippocampus, apoE4 mice displaying an hyper-activation state of Rho proteins. In this context, it has been shown that an excessive Rho activity, negatively affects synaptic and cognitive functions [42] and errors in cellular modulators of APP processing induced by polymorphisms (such as the apoE4 ε-allele) predisposes an individual to early or late-onset AD induced by an hyper-activation of the Rho family GTPases [43]. Our results demonstrate that CNF1 is able to switch off the hyper-activation of Rho proteins in the hippocampus and the mechanism by which CNF1 counteracts this phenomenon most probably involves the ubiquitin-mediated proteasomal degradation of activated Rho GTPases. The involvement of the ubiquitin-proteasome pathway in CNF1 activity was first reported by Doye and coworkers in 2002 [23], and subsequently confirmed by several other authors [69], [70]. All studies so far conducted on this matter have been performed in cell cultures but, obviously, the in vivo situation is much more complex. In fact, there is not only a genotype-dependent difference in terms of Rho/Rac activation (this difference is also reinforced by the diverse expression of inflammatory markers IL-1β and Beta-amyloid) but also there is a difference between the two brain areas. This is a well known phenomenon, hippocampus and cortex differing in term of neurotransmitter dynamics, structures and plasticity [71] and our results highlight a different Rho GTPases activation state by CNF1 in the two brain areas, CNF1 decreasing Rho proteins' activation in the hippocampus while activating them in the frontal cortex. This is probably due to the fact that CNF1 most certainly stimulates the activation/degradation process of Rho GTPases in both areas, but with a different outcome depending on initial activation status of Rho proteins. It is also relevant that CNF1 increases ATP availability in both hippocampus and cortex of apoE4 mice, although at different extent. How RhoA and Rac1 signaling can increase ATP is still uncharted and under investigation by our group, but we can hypothesize that the increase in ATP content observed in both brain regions could probably be linked to the CNF1-induced activation/degradation process. Furthermore, we have previously reported that CNF1 influences the mitochondrial homeostasis [72], induces a remarkable modification in the mitochondrial network architecture, with the appearance of elongated and interconnected mitochondria [50], and promotes an increment of proteins such as creatine and phosphocreatine, which are involved in ATP regeneration [73] in the brain of pathological murine models [20]. All these effects persist for long periods of time in mouse brains (as shown in [20], the brain mitochondrial activity is still increased 90 days after CNF1 injection), suggesting the persistence of the CNF1 molecular effects rather the persistence of the toxin in the CNS. On this basis, mitochondria may be regarded as one of the target cell organelle of CNF1, this toxin playing an important role for the restoration of apoE4 brain energy balance. Furthermore, such an increase in ATP content could promote the amelioration of cognitive functions observed in apoE4 mice, where the hippocampal synaptic plasticity is altered by Aβ accumulation [74]. In this context, it is interesting to note that in apoE4 hippocampus the CNF1-dependent decrease of Rho and Rac, which occurs with a very significative increment of ATP content, is accompanied by counteraction of important neuroinflammatory markers of AD, such as Aβ deposition and IL-1β overexpression. It is known that ATP as well as other high-energy compounds, potently increase APP α-processing of β-amyloid precursor protein thus leading to a decrease in Aβ [75]. On the other hand, it is well established that inflammatory cytokines and Aβ are intimately related and during early AD pathogenesis, Aβ and IL-1β could bring about a vicious cycle of Aβ generation between astrocytes and neurons leading to chronic, sustained and progressive neuroinflammation [76]. Therefore, the decrease of β-amyloid induced by CNF1 in our experimental system is in line with the corresponding decrease in IL-1β, one of the most important inflammatory mediators in AD. We cannot rule out the possibility, however, that the decrease in Aβ observed in hippocampus is due, at least in part, to the decrease in Rho and Rac activity due to CNF1 in such brain area. It is known, in fact, that inhibition of Rho, as well as of Rac, reduces Aβ production [43]. This hypothesis is also supported by the fact that CNF1 does not change the Aβ level in cortex of CNF1-treated mice, where the balance of CNF1 activity is in favor of a final result of Rho and Rac activation. The few changes in frontal cortex herein described, respect to the hippocampus, however, are also in agreement with the idea of an increased susceptibility of hippocampal neurons to the pathological mechanisms behind some dementias, such as AD [77].

It is interesting to note that the molecular results herein discussed are paralleled by a significant increment of cognitive performances in apoE4 mice, which upon challenge with CNF1 behave quite similarly to apoE3. Therefore, we can speculate that the striking improvement of the cognitive defects in CNF1-treated mice is most probably linked, via the pharmacological modulation of Rho GTPase signaling, to a restoration of physiological energy levels and to anti-inflammatory processes, endorsing CNF1 as a potential therapy against AD, atherosclerosis and neuroinflammation diseases in general.

## Materials and Methods

### CNF1 preparation and treatments

CNF1 was obtained from the 392 ISS strain (kindly provided by V. Falbo, Rome, Italy) and purified essentially as previously described [78] with few modifications in the procedure. For all experiments, a concentration of 0.1 nM CNF1 was used.

### In vivo experiments

#### Experimental animals

Human apoE Target Replacement (TR) Model mice expressing human apoE3 and apoE4, under the control of the murine apoE regulatory sequences and on the C57BL/6J background were used. ApoE3 and apoE4 TR mice were purchased by Taconic Farms (USA) (Homozygous B6.129P2-Apoetm3(APOE*4)Mae N8 and Homozygous B6.129P2-Apoetm2(APOE*3)Mae N8). The model was created by targeting the murine apoE gene for replacement with the human APOE4 and APOE3 allele in E14TG2a ES cells and injecting the targeted cells into blastocysts. Resultant chimeras were backcrossed to C57BL/6 for seven generations (N7). The mice were backcrossed once more (N8) and embryo transfer derived. The colony was maintained through mating homozygotes. Four apoE4 and apoE3 male and eight apoE4 and ApoE3 female mice were bought and the colony was maintained by homozygous breeding. Research Breeding Agreement was delivered by Taconic Farms. All experiments were performed on 12 months old apoE3 TR and apoE4 TR mice.

Mice were housed in groups of 6 in individually micro isolation cage rack (Tecniplast, Italia) with water and food laboratory chow ad libitum and controlled environmental conditions of light (from 7.00 a.m. to 7.00 p.m.), temperature (22±2°C) and humidity (65%).

#### Ethics Statement

Animal experiments were carried out in the Department of Pharmacology of the University of Bologna with the approval of the local ethical committee (Veterinary Service of the University of Bologna) and in agreement with the National Animal Welfare Act. All efforts were made to minimize animal suffering and the number of animals used was kept to a minimum by the experimental design. All the procedures followed in this work were in compliance with the European Community Council Directive of 24 November 1986 (86/609/EEC) and were approved by the Ethical committee of the University of Bologna (05/70/12).

#### Animals surgery

Mice were anaesthetised with Fluoxethane (2% Fluoxethane, air flow 1.8 l/min, Ugo Basile gas anesthesia) and placed in a stereotaxic apparatus KopfAfter general anesthesia (2% Fluoxethane, air flow 1.8 l/minUgo Basile gas anesthesia). Skuls were exposed and a needle connected to a 10-μl Hamilton microsyringe was placed in the right lateral ventricle of the right cerebral hemisphere with a stereotactic technique (Final injection coordinates: AP 0.1 mm, L ±0.9 mm V −2.1 mm from bregma [79]. The Hamilton siringe was connected to a micropump set at a flow-rate of 0.5 ml/min. Two minutes after the injection, the needle was removed and the surgical wound was sutured. The mice returned to their cages for recovery and their conditions were monitored for a 10-day period. Experiments started at 10 and 20 days after surgery. At the end of the behavioral test, animals were sacrified by cerebral dislocation, brains were quickly removed and brain areas of interest were dissected on cold dish and snap-frozen on dry-ice, and stored at −70°C until use.

### Behavioral tasks

#### Water Morris Test

Mice were trained in the MWM task to locate a hidden escape platform in a circular pool [80]. The apparatus consisted of a large circular water tank (1.00 m diameter, 50 cm height) with a transparent round escape platform (10 cm^2^). The pool was virtually divided into four equal quadrants identified as north, east, northwest, southeast, and southwest. The tank was filled with tap water at a temperature of 22±2°C up to 0.5 cm above the top of the platform and the water was made opaque with milk. The platform was placed in the tank in a fixed position (in the middle of the south-east quadrant). The pool was placed in a large room with a number of intra- (squares, triangles, circles and star) and extra-maze visual. After the training, each mouse was tested for 4 trials a day, for 4 consecutive days with an inter-trial interval of 30 min (Acquisition phase). A video camera was placed above the center of the pool and connected to a video-tracking system: Ethovision 3.1© (Noldus Information Technology B.V., Wageningen, Netherlands). Mice were released facing the wall of the pool from one of following starting points: North, East, South, or West and allowed to search for up to 60 s for the platform. If a mouse did not find the platform, it was gently guided to it and allowed to remain there for 15 s. Reference memory was assessed with one trial (Probe trial), on the fifth day, 24 h after the last acquisition trial, using one starting point for all the mice. Mice were allowed to search for up to 30 s for the platform. The latency to find the hidden platform was used as a measure of learning. For the Probe trial, the % time spent in the quadrant in which the platform had been located during training, mean proximity to platform and time to the first entrance in SE quadrant were employed as a measure of retention of acquired spatial preference. The average swim speeds, were also analyzed. All experimental sessions were carried out between 09.00 and 15.00 h.

#### Passive Avoidance

Emotional learning and non-spatial memory were assessed in a passive avoidance test. The instrument consists of a tilting-floor box (47x18x26 (h) cm) divided into two compartments by a sliding door and a control unit incorporating a scrambler shocker (Ugo Basile, Italy). This classic instrument for Pavlovian conditioning exploits the tendency in mice to escape from an illuminated area into a dark one (step-through method). The firsts day mice were individually placed into the illuminated compartment. After 60-seconds of acclimation period, the connecting door between the chambers opened. In general, mice step quickly through the gate and enter the dark compartment because mice prefer to be in the dark. Upon entering the dark compartment, the mice received a brief foot shock (0.3 mA for 3 seconds) and were immediately removed from the chamber. If the mouse remained in the light compartment for the duration of the trial (300 seconds), the door closed and the mouse was removed from the light compartment. The chambers were cleaned with 70% ethanol between testing of individual mice. After a 24 hours retention period, the mice were placed back into the light compartment and the time to re-enter the dark compartment (latency) was measured up to 300 seconds. No shock was administered during the testing phase if the mouse entered the dark compartment before 300 seconds had elapsed. The latency time to re-enter the dark compartment 24 h later was measured.

#### Elevated Plus Maze

Mice were placed in the experimental room 1 h before the test. The apparatus consisted of two open arms, two enclosed arms of the same size (30×8 cm), and a central area (8×8 cm); placement was 50 cm above the floor. At the beginning of a session, mice were placed in the central part of the maze facing one of the open arms. The number of entries and the time spent in the open and close arms were recorded for 5 min. An entry was defined as the mice entering into an arm with all four paws. The test was run out between 09:00 and 15:00 and behavior was videotaped and scored by EthoVision Ethovision 3.0© (Noldus Information Technology B.V., Wageningen, Netherlands).

#### Open Field

Mice were placed in the experimental room 1 h before the test. Open field activity was measured in a square arena (50×50 cm). At the beginning of a session, mice were placed in the central part of the arena. We scored the number of entries into the center, corners, and periphery (corners plus walls) and the time spent in the same areas by video-tracking recording for 20 min. Experiments were carried out under white light between 09:00 and 15:00 h and activity was videotaped and scored by Ethovision 3.0© (Noldus Information Technology B.V., Wageningen, Netherlands).

### Pull down assay

Frozen tissues were homogenized in 50 mM Tris (pH 7.4), 1 mM EDTA (pH 8.0), 0.5% Nonidet P-40, 150 mM NaCl, 10% glycerol, 10 µg/ml aprotinin, 10 µg/ml leupeptin, 1mM PMSF, 1 mM Na_2_VO_4_, and processed for pull-down assay as previously described [23]. The homogenates were clarified by centrifugation at 14,000 rpm for 5 min at 4°C, and incubated with 50 µg of GST-PAK-CD fusion protein (for Rac) bound to glutathione-coupled Sepharose beads (Amersham Pharmacia), and GST-Rhotekin, (Cytoskeleton, for Rho) for 40 min at 4°C. Beads were washed three times in the lysis buffer and bound proteins were eluted in sample buffer, subjected to SDS-PAGE and immunoblotted as described [81]. Whole-cell lysates were analyzed in parallel. Autoradiographs, scanned using the Epson Perfection 1250 scanner, were quantified by means of Image J software and normalized as a function the total proteins loaded in the assay.

### Measurement of ATP

Measurement of tissue ATP was performed using the ATP lite Assay (Perkin Elmer-Cetus, Norwalk, CT, USA). In brief, tissues were omogeneated in 50 µl of lysis buffer and mixed for 10 min. Forty microlitres of substrate solution (Luciferase/Luciferin) was added to each sample. The luminescence was measured using a luminescence plate reader (Victor3-V, PerkinElmer Life Sciences). The ATP concentration was normalized to total tissue protein concentration estimated by Bradford protein assay (Bio-Rad).

### Enzyme-linked immunosorbent assay (ELISA)

Frozen tissues were lysed in ice-cold RIPA buffer (Tris-HCl pH 7.5 150 mM, NaCl 150 mM, glycerol 10%, Triton X-100 1%) containing 10 µg/ml aprotinin, 10 µg/ml leupeptin, 1 mM PMSF, 1 mM Na_2_VO_4_, and homogenated by a dounce homogenizer. Following centrifugation at 14000 rpm for 5 minutes, levels of IL-1β and IL-6 in the supernatants were determined by using microtiter wells plates coated with anti-mouse IL antibodies (BioVendor-Laboratorni, Czeck Republic) according to the manufacturer's instructions.

For Aβ42 measurement, frozen tissues were lysed in 5 M guanidine HCl/50 mM Tris-HCl and homogenated as above described. The homogenates were mixed for 3 hours at room temperature and then diluted 1:10 with cold reaction buffer (Dulbecco's PBS with 5% BSA and 0.03% Tween-20) containing protease inhibitors cocktail (Sigma). Levels of Aβ42 in the samples were determined by mean of Ms Aβ42 kit (Invitrogen), according to the manufacturer's instructions.

### Statistical analysis

Data are presented as mean ± sem. Statistical analysis was performed by means of the general linear model (repeated measures or general factorial) analysis of variance (ANOVA). When a significant interaction was detected, we also performed a Fisher LSD post-hoc test. For probe data one-way ANOVA was used followed by Fisher LSD exsact post-hoc test. *p*<0.05 was considered as a threshold for significant difference.

## Supporting Information

File S1 **Contains Tables S1-S20.** Summary of the statistical analysis.
(DOC)
