# Correction: CNF1 Increases Brain Energy Level, Counteracts Neuroinflammatory Markers and Rescues Cognitive Deficits in a Murine Model of Alzheimer's Disease

**DOI:** 10.1371/annotation/8da0f878-fcab-4f65-bad0-c5bdda8181ed

**Published:** 2013-06-03

**Authors:** Stefano Loizzo, Roberto Rimondini, Sara Travaglione, Alessia Fabbri, Marco Guidotti, Alberto Ferri, Gabriele Campana, Carla Fiorentini

Affiliation 2 is incorrect.

The correct affiliation for Roberto Rimondini is: Medical and Surgical Sciences Department - DIMEC - University of Bologna, Bologna, Italy

The correct affiliation for Gabriele Campana is: Pharmacy and Biotechnology Department - FaBiT - University of Bologna, Bologna, Italy 

