# Supplementary material for: CNF1 Increases Brain Energy Level, Counteracts Neuroinflammatory Markers and Rescues Cognitive Deficits in a Murine Model of Alzheimer's Disease
Source: PLoS One. 2013 May 30;8(5):e65898. doi: 10.1371/journal.pone.0065898 (PMC3667817; doi:10.1371/journal.pone.0065898)
Supplement: File S1 — Contains Tables S1-S20. Summary of the statistical analysis. (DOC) [file pone.0065898.s001.doc]

**Supporting Information**

**Morris Water maze test**

**A) Learning Phase**

**Table S1- Learning**

**Repeated measure Two-way ANOVA**

| **Effect** | **D.F.** | **F** | **p** |
| --- | --- | --- | --- |
| **Time** | 3/141 | 32,57 | <0,001 |
| **Genotype** | 1/50 | 11,47 | 0,0014 |
| **Treatment** | 1/50 | 14,91 | 0,00034 |
| **Time X Gen** | 3/141 | 0,22 | NS |
| **Time X Treat** | 3/141 | 3,06 | 0,03 |
| **Gen X Treat** | 1/50 | 4,72 | 0,035 |
| **Time X Gen X Treat** | 3/141 | 3,42 | 0,018 |

**Table S2- Learning**

**One-way ANOVA**

| **Effect** | **D.F.** | **F** | **p** |
| --- | --- | --- | --- |
| **Main effect** | 12/130 | 3,9276 | 0.00004 |

**B) Memory phase (Probe)**

Statistical analysis regarding 1) % Time spent in former platform quadrant (SE)(S3, S4)), 2) latency to first entrance in SE (S5, S6), 3) mean proximity test (Gallagher’s test)(S7, S8)

**Table S3- Memory (% Time spent in SE)**

**Two-way ANOVA**

| **Effect** | **D.F.** | **F** | **p** |  |
| --- | --- | --- | --- | --- |
| **Genotype** | 1/50 | 0,094 | NS |  |
| **Treatment** | 1/50 | 9,274 | 0,0037 |  |
| **Gen X Treat** | 1/50 | 4,45 | 0,035 |  |

**Table S4.- Memory (% Time spent in SE)**

One-way ANOVA

| **Effect** | **D.F.** | **F** | **p** |
| --- | --- | --- | --- |
| **Main effect** | 3/50 | 54,624 | 0.0064 |

**Table S5- Memory (latency of the first entrance in SE)**

Two-way ANOVA

| **Effect** | **D.F.** | **F** | **p** |  |
| --- | --- | --- | --- | --- |
| **Genotype** | 1/50 | 3,852 | NS |  |
| **Treatment** | 1/50 | 10,339 | 0,002 |  |
| **Gen X Treat** | 1/50 | 19,326 | 0,00006 |  |

**Table S6- Memory (latency of the first entrance in SE)**

One-way ANOVA

| **Effect** | **D.F.** | **F** | **p** |
| --- | --- | --- | --- |
| **Main effect** | 3/50 | 3.0323 | 0.037 |

**Table S7- Memory (mean proximity to former platform location, Gallangher’s proximity test)**

Two-way ANOVA

| **Effect** | **D.F.** | **F** | **p** |  |
| --- | --- | --- | --- | --- |
| **Genotype** | 1/50 | 3,852 | NS |  |
| **Treatment** | 1/50 | 10,339 | 0,002 |  |
| **Gen X Treat** | 1/50 | 19,326 | 0,00006 |  |

**Table S8- Memory (mean proximity to former platform location, Gallangher’s proximity test)**

One-way ANOVA

| **Effect** | **D.F.** | **F** | **p** |
| --- | --- | --- | --- |
| **Main effect** | 3/50 | 4.648 | 0.0068 |

**Table S9- Passive avoidance (test day 2)**

**One-way ANOVA.**

| **Effect** | **D.F.** | **F** | **p** |
| --- | --- | --- | --- |
| **Treatment** | 3/50 | 4,9547 | 0,00434 |

**Table S10- Elevated Plus Maze Total Entries (TE)**

**One-way ANOVA.**

| **Effect** | **D.F.** | **F** | **p** |  |
| --- | --- | --- | --- | --- |
| **Genotype** | 1/50 | 1.3161 | 0.25675 |  |
| **Treatment** | 1/50 | 1.2894 | 0.26157 |  |
| **Gen X Treat** | 1/50 | 1.3868 | 0.24452 |  |

**Table 11- Elevated Plus Maze % open entries (%OE)**

**One-way ANOVA.**

| **Effect** | **D.F.** | **F** | **p** |  |
| --- | --- | --- | --- | --- |
| **Genotype** | 1/50 | 0.34155 | 0.56156 |  |
| **Treatment** | 1/50 | 0.21216 | 0.64708 |  |
| **Gen X Treat** | 1/50 | 0.39574 | 0.53216 |  |

**Table S12- Elevated Plus Maze** **% open time (%OT)**

**One-way ANOVA.**

| **Effect** | **D.F.** | **F** | **p** |  |
| --- | --- | --- | --- | --- |
| **Genotype** | 1/50 | 0.59611 | 0.44370 |  |
| **Treatment** | 1/50 | 1.3952 | 0.24312 |  |
| **Gen X Treat** | 1/50 | 0.38472 | 0.53790 |  |

**Table S13- ATP (hippocampus)**

**Two -way ANOVA.**

| **Effect** | **D.F.** | **F** | **p** |  |
| --- | --- | --- | --- | --- |
| **Genotype** | 1/31 | 4.3068 | 0.0463 |  |
| **Treatment** | 1/31 | 17.004 | 0.00026 |  |
| **Gen X Treat** | 1/31 | 9.274 | 0.004 |  |

**Table S14- ATP (cortex)**

**Two -way ANOVA.**

| **Effect** | **D.F.** | **F** | **p** |  |
| --- | --- | --- | --- | --- |
| **Genotype** | 1/20 | 0.0298 | 0.8 |  |
| **Treatment** | 1/20 | 22.685 | 0.00012 |  |
| **Gen X Treat** | 1/20 | 4.824 | 0.04001 |  |

**Table S15- IL-1β (hippocampus)**

**Two -way ANOVA.**

| **Effect** | **D.F.** | **F** | **p** |  |
| --- | --- | --- | --- | --- |
| **Genotype** | 1/22 | 25.727 | 0.00004 |  |
| **Treatment** | 1/22 | 5,8514 | 0.024 |  |
| **Gen X Treat** | 1/22 | 25,757 | 0.00004 |  |

**Table S16- IL-1β (cortex)**

**Two -way ANOVA.**

| **Effect** | **D.F.** | **F** | **p** |  |
| --- | --- | --- | --- | --- |
| **Genotype** | 1/20 | 0.37281 | 0.54836 |  |
| **Treatment** | 1/20 | 0.20636 | 0.65453 |  |
| **Gen X Treat** | 1/20 | 1.6018 | 0.22019 |  |

**Table S17- IL-6 (hippocampus)**

**Two -way ANOVA.**

| **Effect** | **D.F.** | **F** | **p** |  |
| --- | --- | --- | --- | --- |
| **Genotype** | 1/21 | 0.35621 | 0.55700 |  |
| **Treatment** | 1/21 | 11,204 | 0.00305 |  |
| **Gen X Treat** | 1/21 | 2,6057 | 0.12141 |  |

**Table S18- IL-6 (cortex)**

**Two -way ANOVA.**

| **Effect** | **D.F.** | **F** | **p** |  |
| --- | --- | --- | --- | --- |
| **Genotype** | 1/20 | 0.05564 | 0.81592 |  |
| **Treatment** | 1/20 | 0.40071 | 0.53389 |  |
| **Gen X Treat** | 1/20 | 3.4200 | 0.07925 |  |

**Table S19- Aβ (hippocampus)**

**Two -way ANOVA.**

| **Effect** | **D.F.** | **F** | **p** |  |
| --- | --- | --- | --- | --- |
| **Genotype** | 1/21 | 0.94182 | 0.34286 |  |
| **Treatment** | 1/21 | 2.0886 | 0.16316 |  |
| **Gen X Treat** | 1/21 | 15.255 | 0.00081 |  |

**Table S20- Aβ (cortex)**

**Two -way ANOVA.**

| **Effect** | **D.F.** | **F** | **p** |  |
| --- | --- | --- | --- | --- |
| **Genotype** | 1/20 | 0.3309 | 0.571 |  |
| **Treatment** | 1/20 | 1.8071 | 0.1939 |  |
| **Gen X Treat** | 1/20 | 0.212 | 0.649 |  |
